# A new bound on the block restricted isometry constant in compressed sensing

**DOI:** 10.1186/s13660-017-1448-2

**Published:** 2017-08-01

**Authors:** Yi Gao, Mingde Ma

**Affiliations:** 1 School of Mathematics and Information Science, Beifang University of Nationalities, Wenchang Road, Yinchuan, 750021 China; 2 Editorial Department of University Journal, Beifang University of Nationalities, Wenchang Road, Yinchuan, 750021 China

**Keywords:** compressed sensing, $l_{2}/l_{1}$-minimization, block sparse recovery, block restricted isometry property, null space property

## Abstract

This paper focuses on the sufficient condition of block sparse recovery with the $l_{2}/l_{1}$-minimization. We show that if the measurement matrix satisfies the block restricted isometry property with $\delta_{2s|\mathcal{I}}< 0.6246$, then every block *s*-sparse signal can be exactly recovered via the $l_{2}/l_{1}$-minimization approach in the noiseless case and is stably recovered in the noisy measurement case. The result improves the bound on the block restricted isometry constant $\delta_{2s|\mathcal {I}}$ of Lin and Li (Acta Math. Sin. Engl. Ser. 29(7):1401-1412, 2013).

## Introduction

Compressed sensing [2–4] is a scheme which shows that some signals can be reconstructed from fewer measurements compared to the classical Nyquist-Shannon sampling method. This effective sampling method has a number of potential applications in signal processing, as well as other areas of science and technology. Its essential model is

1 $$ \min_{x \in\mathbb{R}^{N}}\|x\|_{0} \quad \textit{s.t.} \quad y=Ax, $$

where $\|x\|_{0}$ denotes the number of non-zero entries of the vector *x*, an *s*-sparse vector $x \in\mathbb{R}^{N}$ is defined by $\|x\|_{0}\leq s \ll N$. However, the $l_{0}$-minimization (1) is a nonconvex and NP-hard optimization problem [5] and thus is computationally infeasible. To overcome this problem, one proposed the $l_{1}$-minimization [4, 6–9].

2 $$ \min_{x \in\mathbb{R}^{N}}\|x\|_{1} \quad \textit{s.t.}\quad y=Ax, $$

where $\|x\|_{1}=\sum_{i=1}^{N}|x_{i}|$. Candès [10] proved that the solutions to (2) are equivalent to those of (1) provided that the measurement matrices satisfy the restricted isometry property (RIP) [9, 11] with some definite restricted isometry constant (RIC) $\delta_{s} \in(0,1)$, here $\delta_{s} $ is defined as the smallest constant satisfying

3 $$ (1-\delta_{s})\|x\|^{2}_{2}\leq\|Ax \|^{2}_{2}\leq(1+\delta_{s})\|x \|^{2}_{2} $$

for any *s*-sparse vectors $x \in\mathbb{R}^{N}$.

However, the standard compressed sensing only considers the sparsity of the recovered signal, but it does not take into account any further structure. In many practical applications, for example, DNA microarrays [12], face recognition [13], color imaging [14], image annotation [15], multi-response linear regression [16], etc., the non-zero entries of sparse signal can be aligned or classified into blocks, which means that they appear in regions in a regular order instead of arbitrarily spread throughout the vector. These signals are called the block sparse signals and has attracted considerable interests; see [17–23] for more information.

Suppose that $x\in\mathbb{R}^{N}$ is split into *m* blocks, $x[1],x[2],\ldots,x[m]$, which are of length $d_{1}, d_{2},\ldots,d_{m}$, respectively, that is,

4 $$ x=[\underbrace{x_{1},\ldots,x_{d_{1}}}_{x[1]}, \underbrace {x_{d_{1}+1},\ldots,x_{d_{1}+d_{2}}}_{x[2]},\ldots, \underbrace{x_{N-d_{m}+1},\ldots,x_{N}}_{x[m]}]^{T}, $$

and $N=\sum_{i=1}^{m}d_{i}$. A vector $x\in\mathbb{R}^{N}$ is called block *s*-sparse over $\mathcal {I}=\{d_{1},d_{2},\ldots,d_{m}\}$ if $x[i]$ is non-zero for at most *s* indices *i* [18]. Obviously, $d_{i}=1$ for each *i*, the block sparsity reduces to the conventional definition of a sparse vector. Let

$$ \|x\|_{2,0}=\sum_{i=1}^{m}I\bigl( \big\| x[i]\big\| _{2}\bigr), $$

where $I(x)$ is an indicator function that equals 1 if $x>0$ and 0 otherwise. So a block *s*-sparse vector *x* can be defined by $\|x\|_{2,0}\leq s$, and $\|x\|_{0}=\sum_{i=1}^{m}\|x[i]\|_{0}$. Also, let $\Sigma_{s}$ denote the set of all block *s*-sparse vectors: $\Sigma_{s}=\{x\in\mathbb{R}^{N}:\|x\|_{2,0}\leq s \}$.

To recover a block sparse signal, similar to the standard $l_{0}$-minimization, one seeks the sparsest block sparse vector via the following $l_{2}/l_{0}$-minimization [13, 17, 18]:

5 $$ \min_{x \in\mathbb{R}^{N}}\|x\|_{2,0} \quad \textit{s.t.} \quad y=Ax. $$

But the $l_{2}/l_{0}$-minimization problem is also NP-hard. It is natural to use the $l_{2}/l_{1}$-minimization to replace the $l_{2}/l_{0}$-minimization [13, 17, 18, 24].

6 $$ \min_{x \in\mathbb{R}^{N}}\|x\|_{2,1} \quad \textit{s.t.} \quad y=Ax, $$

where

7 $$ \|x\|_{2,1}= \sum_{i=1}^{m}\big\| x[i] \big\| _{2}. $$

To characterize the performance of this method, Eldar and Mishali [18] proposed the block restricted isometry property (block RIP).

### Definition 1

Block RIP

Given a matrix $A \in\mathbb{R}^{n\times N}$, for every block *s*-sparse $x\in\mathbb{R}^{N}$ over $\mathcal{I}=\{d_{1},d_{2},\ldots,d_{m}\}$, there exists a positive constant $0<\delta_{s|\mathcal{I}}<1$, such that

8 $$ (1-\delta_{s|\mathcal{I}})\|x\|^{2}_{2}\leq\|Ax \|^{2}_{2}\leq(1+\delta _{s|\mathcal{I}})\|x \|^{2}_{2}, $$

then the matrix *A* satisfies the *s*-order block RIP over $\mathcal {I}$, and the smallest constant $\delta_{s|\mathcal{I}}$ satisfying the above inequality (8) is called the block RIC of *A*.

Obviously, the block RIP is an extension of the standard RIP, but it is a less stringent requirement comparing to the standard RIP [18, 25]. Eldar *et al.* [18] proved that the $l_{2}/l_{1}$-minimization can exactly recover any block *s*-sparse signal when the measurement matrices *A* satisfy the block RIP with $\delta_{2s|\mathcal{I}}<0.414$. The block RIC can be improved, for example, Lin and Li [1] improved the bound to $\delta_{2s|\mathcal{I}}<0.4931$, and established another sufficient condition $\delta_{s|\mathcal {I}}<0.307$ for exact recovery. So far, to the best of our knowledge, there is no paper that further focuses on improvement of the block RIC. As mentioned in [1, 26, 27], like RIC, there are several benefits for improving the bound on $\delta_{2s|\mathcal{I}}$. First, it allows more measurement matrices to be used in compressed sensing. Secondly, for the same matrix *A*, it allows for recovering a block sparse signal with more non-zero entries. Furthermore, it gives better error estimation in a general problem to recover noisy compressible signals. Therefore, this paper addresses improvement of the block RIC, we consider the following minimization for the inaccurate measurement, $y=Ax+e$ with $\|e\|_{2} \leq\epsilon$:

9 $$ \min_{x \in\mathbb{R}^{N}}\|x\|_{2,1} \quad \textit{s.t.} \quad \|y-Ax\|_{2}\leq\epsilon. $$

Our main result is stated in the following theorem.

### Theorem 1

*Suppose that the* 2*s block RIC of the matrix* $A\in\mathbb{R}^{n\times N}$ *satisfies*

10 $$ \delta_{2s|\mathcal{I}}< \frac{4}{\sqrt{41}}\approx0.6246. $$

*If* $x^{\ast}$ *is a solution to* (9), *then there exist positive constants* $C_{1}$, $D_{1}$ *and* $C_{2}$, $D_{2}$, *and we have*

11 $$\begin{aligned}& \big\| x-x^{\ast}\big\| _{2,1}\leq C_{1} \sigma_{s}(x)_{2,1}+D_{1} \sqrt {s}\epsilon, \end{aligned}$$

12 $$\begin{aligned}& \big\| x-x^{\ast}\big\| _{2} \leq\frac{C_{2}}{\sqrt{s}} \sigma_{s}(x)_{2,1} + D_{2} \epsilon, \end{aligned}$$

*where the constants* $C_{1}$, $D_{1}$ *and* $C_{2}$, $D_{2}$ *depend only on* $\delta _{2s|\mathcal{I}}$, *written as*

13 $$\begin{aligned}& C_{1}= \frac{2(4\sqrt{1-\delta_{2s|\mathcal{I}}^{2}}+3\delta_{2s|\mathcal {I}})}{4\sqrt{1-\delta_{2s|\mathcal{I}}^{2}}-5\delta_{2s|\mathcal{I}}}, \end{aligned}$$

14 $$\begin{aligned}& D_{1}= \frac{16\sqrt{1+\delta_{2s|\mathcal{I}}}}{4\sqrt{1-\delta _{2s|\mathcal{I}}^{2}}-5\delta_{2s|\mathcal{I}}}, \end{aligned}$$

15 $$\begin{aligned}& C_{2}=\frac{2(4\sqrt{1-\delta_{2s|\mathcal{I}}^{2}}+3\delta_{2s|\mathcal{I}})^{2}}{ (4\sqrt{1-\delta_{2s|\mathcal{I}}^{2}}-\delta_{2s|\mathcal{I}})(4\sqrt {1-\delta_{2s|\mathcal{I}}^{2}}-5\delta_{2s|\mathcal{I}})}, \end{aligned}$$

16 $$\begin{aligned}& D_{2}=\frac{8\sqrt{1+\delta_{2s|\mathcal{I}}}(12\sqrt{1-\delta _{2s|\mathcal{I}}^{2}}+\delta_{2s|\mathcal{I}})}{ (4\sqrt{1-\delta_{2s|\mathcal{I}}^{2}}-\delta_{2s|\mathcal{I}})(4\sqrt {1-\delta_{2s|\mathcal{I}}^{2}}-5\delta_{2s|\mathcal{I}})}, \end{aligned}$$

*and* $\sigma_{s}(x)_{2,1}$ *denotes the best block* *s*-*term approximation error of* $x \in\mathbb{R}^{N}$ *in* $l_{2}/l_{1}$ *norm*, *i*.*e*.,

17 $$ \sigma_{s}(x)_{2,1}:=\inf_{z\in\Sigma_{s}}\|x-z \|_{2,1}. $$

### Corollary 1

*Under the same assumptions as in Theorem* 1, *suppose that* $e=0$ *and* *x* *is block* *s*-*sparse*, *then* *x* *can be exactly recovered via the* $l_{2}/l_{1}$-*minimization* (6).

The remainder of the paper is organized as follows. In Section 2, we introduce the $l_{2,1}$ robust block NSP that can characterize the stability and robustness of the $l_{1}$-minimization with noisy measurement (9). In Section 3, we show that the condition (10) can conclude the $l_{2,1}$ robust block NSP, which means to implement the proof of our main result. Section 4 is for our conclusions. The last section is an appendix including an important lemma.

## Block null space property

Although null space property (NSP) is a very important concept in approximation theory [28, 29], it provides a necessary and sufficient condition of the existence and uniqueness of the solution to the $l_{1}$-minimization (2), so NSP has drawn extensive attention for studying the characterization of measurement matrix in compressed sensing [30]. It is natural to extend the classic NSP to the block sparse case. For this purpose, we introduce some notations. Suppose that $x\in\mathbb{R}^{N}$ is an *m*-block signal, whose structure is like (4), we set $S\subset\{1,2,\ldots,m\}$ and by $S^{C}$ we mean the complement of the set *S* with respect to $\{1,2,\ldots,m\}$, *i.e.*, $S^{C}=\{1,2,\ldots,m\}\setminus S$. Let $x_{S}$ denote the vector equal to *x* on a block index set *S* and zero elsewhere, then $x=x_{S} + x_{S^{C}}$. Here, to investigate the solution to the model (9), we introduce the $l_{2,1}$ robust block NSP, for more information on other forms of block NSP, we refer the reader to [23, 31].

### Definition 2

$l_{2,1}$ robust block NSP

Given a matrix $A \in\mathbb{R}^{n\times N}$, for any set $S\subset\{ 1,2,\ldots,m\}$ with $\operatorname{card}(S)\leq s$ and for all $v \in\mathbb{R}^{N}$, if there exist constants $0< \tau< 1$ and $\gamma>0$, such that

18 $$ \|v_{S}\|_{2} \leq\frac{\tau}{\sqrt{s}} \|v_{S^{C}}\|_{2,1}+ \gamma\|Av\|_{2}, $$

then the matrix *A* is said to satisfy the $l_{2,1}$ robust block NSP of order *s* with *τ* and *γ*.

Our main result relies heavily on this definition. A natural question is what relationship between this robust block NSP and the block RIP. Indeed, from the next section, we shall see that the block RIP with condition (10) can lead to the $l_{2,1}$ robust block NSP, that is, the $l_{2,1}$ robust block NSP is weaker than the block RIP to some extent. The spirit of this definition is first to imply the following theorem.

### Theorem 2

*For any set* $S\subset\{1,2,\ldots,m\}$ *with* $\operatorname{card}(S)\leq s$, *the matrix* $A \in\mathbb{R}^{n \times N}$ *satisfies the* $l_{2,1}$ *robust block NSP of order* *s* *with constants* $0< \tau< 1$ *and* $\gamma>0$, *then*, *for all vectors* $x,z \in\mathbb{R}^{N}$,

19 $$ \|x-z\|_{2,1}\leq \frac{1+\tau}{1-\tau}\bigl(\|z\|_{2,1}- \|x\|_{2,1} + 2\|x_{S^{C}}\|_{2,1}\bigr) + \frac{2\gamma\sqrt{s}}{1-\tau} \big\| A(x-z)\big\| _{2}. $$

### Proof

For $x,z \in\mathbb{R}^{N}$, setting $v=x-z$, we have

$$\begin{aligned}& \|v_{S^{C}}\|_{2,1} \leq\|x_{S^{C}}\|_{2,1}+ \|z_{S^{C}}\|_{2,1}, \\& \|x\|_{2,1}= \|x_{S^{C}}\|_{2,1}+\|x_{S} \|_{2,1} \leq\|x_{S^{C}}\|_{2,1}+\|v_{S} \|_{2,1}+\|z_{S}\|_{2,1}, \end{aligned}$$

which yield

20 $$ \|v_{S^{C}}\|_{2,1}\leq2\|x_{S^{C}} \|_{2,1}+\|z\|_{2,1}-\|x\|_{2,1}+\| v_{S} \|_{2,1}. $$

Clearly, for an *m*-block vector $x\in\mathbb{R}^{N}$ is like (4), $l_{2}$-norm $\|x\|_{2}$ can be rewritten as

21 $$ \|x\|_{2}=\|x\|_{2,2}= \sum_{i=1}^{m} \bigl(\big\| x[i]\big\| _{2}^{2}\bigr)^{1/2}. $$

Thus, we have $\|v_{S}\|_{2,2}=\|v_{S}\|_{2}$ and $\|v_{S}\|_{2,1}\leq\sqrt {s}\|v_{S}\|_{2,2}$. So the $l_{2,1}$ robust block NSP implies

22 $$ \|v_{S}\|_{2,1}\leq\tau\|v_{S^{C}} \|_{2,1}+\gamma\sqrt{s}\|Av\|_{2}. $$

Combining (20) with (22), we can get

$$ \|v_{S^{C}}\|_{2,1} \leq \frac{1}{1-\tau}\bigl(2\|x_{S^{C}} \|_{2,1}+\|z\| _{2,1}-\|x\|_{2,1}\bigr) +\frac{\gamma\sqrt{s}}{1-\tau} \|Av\|_{2}. $$

Using (22) once again, we derive

$$\begin{aligned} \|v \|_{2,1}&=\|v_{S^{C}}\|_{2,1}+\|v_{S} \|_{2,1} \leq(1+\tau)\| v_{S^{C}}\|_{2,1}+\gamma\sqrt{s} \|Av\|_{2} \\ &\leq\frac{1+\tau}{1-\tau}\bigl(2\|x_{S^{C}}\|_{2,1}+\|z \|_{2,1}-\|x\|_{2,1}\bigr) +\frac{2\gamma\sqrt{s}}{1-\tau}\|Av\|_{2}, \end{aligned}$$

which is the desired inequality. □

The $l_{2,1}$ robust block NSP is vital to characterize the stability and robustness of the $l_{2}/l_{1}$-minimization with noisy measurement (9), which is the following result.

### Theorem 3

*Suppose that the matrix* $A\in\mathbb{R}^{n\times N}$ *satisfies the* $l_{2,1}$ *robust block NSP of order* *s* *with constants* $0< \tau<1$ *and* $\gamma>0$, *if* $x^{\ast}$ *is a solution to the* $l_{2}/l_{1}$-*minimization with* $y=Ax+e$ *and* $\|e\|_{2}\leq\epsilon$, *then there exist positive constants* $C_{3}$, $D_{3}$ *and* $C_{4}$, $D_{4}$, *and we have*

23 $$\begin{aligned}& \big\| x-x^{\ast}\big\| _{2,1}\leq C_{3} \sigma_{s}(x)_{2,1}+D_{3} \sqrt {s}\epsilon, \end{aligned}$$

24 $$\begin{aligned}& \big\| x-x^{\ast}\big\| _{2} \leq\frac{C_{4}}{\sqrt{s}} \sigma_{s}(x)_{2,1} + D_{4} \epsilon, \end{aligned}$$

*where*

25 $$\begin{aligned}& C_{3}= \frac{2(1+\tau)}{1-\tau};\qquad D_{3}= \frac{4\gamma}{1-\tau}. \end{aligned}$$

26 $$\begin{aligned}& C_{4}= \frac{2(1+\tau)^{2}}{1-\tau};\qquad D_{4}= \frac{2\gamma(3+\tau)}{1-\tau}. \end{aligned}$$

### Proof

In Theorem 2, by *S* denote an index set of *s* largest $l_{2}$-norm terms out of *m* blocks in *x*, (23) is a direct corollary of Theorem 2 if we notice that $\|x_{S^{C}}\|_{2,1}=\sigma_{s}(x)_{2,1}$ and $\|A(x-x^{\ast})\|_{2} \leq 2\epsilon$. Equation (24) is a result of Theorem 7 for $q=1$ in [23]. □

## Proof of the main result

From Theorem 3, we see that the inequalities (23) and (24) are the same as in (11) and (12) up to constants, respectively. This means that we shall only show that the condition (10) implies the $l_{2,1}$ robust block NSP for implementing the proof of our main result.

### Theorem 4

*Suppose that the* 2*s block RIC of the matrix* $A\in\mathbb{R}^{n\times N}$ *obeys* (10), *then the matrix* *A* *satisfies the* $l_{2,1}$ *robust block NSP of order* *s* *with constants* $0< \tau<1$ *and* $\gamma>0$, *where*

27 $$ \tau=\frac{4\delta_{2s|\mathcal{I}}}{4\sqrt{1-\delta_{2s|\mathcal {I}}^{2}}-\delta_{2s|\mathcal{I}}},\qquad \gamma=\frac{4\sqrt{1+\delta_{2s|\mathcal{I}}}}{4\sqrt{1-\delta _{2s|\mathcal{I}}^{2}}-\delta_{2s|\mathcal{I}}}. $$

### Proof

The proof relies on a technique introduced in [30]. Suppose that the matrix *A* has the block RIP with $\delta_{2s|\mathcal{I}}$. Let *v* be divided into *m* blocks whose structure is like (38). Let $S=:S_{0}$ be an index set of *s* largest $l_{2}$-norm terms out of *m* blocks in *v*. We begin by dividing $S^{C}$ into subsets of size *s*, $S_{1}$ is the first *s* largest $l_{2}$-norm terms in $S^{C}$, $S_{2}$ is the next *s* largest $l_{2}$-norm terms in $S^{C}$, etc. Since the vector $v_{S}$ is block *s*-sparse, according to the block RIP, for $|t|\leq\delta_{2s|\mathcal{I}}$, we can write

28 $$ \|Av_{S}\|_{2}^{2}=(1+t) \|v_{S}\|_{2}^{2}. $$

We are going to establish that, for any $j\geq1$,

29 $$ \big|\langle Av_{S}, Av_{S_{j}}\rangle\big| \leq\sqrt {\delta_{2s|\mathcal {I}}^{2}-t^{2}}\|v_{S} \|_{2}\|v_{S_{j}}\|_{2}. $$

To do so, we normalize the vectors $v_{S}$ and $v_{S_{j}}$ by setting $u=:v_{S}/\|v_{S}\|_{2}$ and $w=:v_{S_{j}}/\|v_{S_{j}}\|_{2}$. Then, for $\alpha, \beta>0$, we write

30 $$ 2\langle Au, Aw\rangle = \frac{1}{\alpha+\beta} \bigl[\big\| A(\alpha u + w) \big\| _{2}^{2}-\big\| A(\beta u - w)\big\| _{2}^{2}- \bigl(\alpha^{2}-\beta^{2}\bigr)\|Au\|_{2}^{2} \bigr]. $$

By the block RIP, on the one hand, we have

$$\begin{aligned} 2\langle Au, Aw\rangle \leq{}& \frac{1}{\alpha+\beta} \bigl[(1+\delta_{2s|\mathcal{I}}) \| \alpha u + w\|_{2}^{2} -(1-\delta_{2s|\mathcal{I}})\|\beta u - w\|_{2}^{2} \bigr] \\ &- \frac{1}{\alpha+\beta}\bigl(\alpha^{2}-\beta^{2}\bigr) (1+t)\|u\|_{2}^{2} \\ ={} & \frac{1}{\alpha+\beta} \bigl[\alpha^{2}(\delta_{2s|\mathcal{I}}-t)+ \beta^{2}(\delta_{2s|\mathcal{I}}+t)+2\delta_{2s|\mathcal{I}} \bigr]. \end{aligned}$$

Making the choice $\alpha=\frac{(\delta_{2s|\mathcal{I}}+t)}{\sqrt {\delta_{2s|\mathcal{I}}^{2}-t^{2}}}$, $\beta=\frac{(\delta_{2s|\mathcal{I}}-t)}{\sqrt{\delta_{2s|\mathcal {I}}^{2}-t^{2}}}$, we derive

31 $$ \langle Au, Aw\rangle\leq\sqrt{\delta_{2s|\mathcal{I}}^{2}-t^{2}}. $$

On the other hand, we also have

$$ \begin{aligned} 2\langle Au, Aw\rangle \geq{}& \frac{1}{\alpha+\beta} \bigl[(1-\delta_{2s|\mathcal{I}}) \| \alpha u + w\|_{2}^{2} -(1+\delta_{2s|\mathcal{I}})\|\beta u - w\|_{2}^{2} \bigr] \\ & - \frac{1}{\alpha+\beta}\bigl(\alpha^{2}-\beta^{2}\bigr) (1+t)\|u\|_{2}^{2} \\ ={} & {-}\frac{1}{\alpha+\beta} \bigl[\alpha^{2}(\delta_{2s|\mathcal{I}}-t)+ \beta^{2}(\delta_{2s|\mathcal{I}}+t)+2\delta_{2s|\mathcal{I}} \bigr]. \end{aligned}$$

Making the choice $\alpha=\frac{(\delta_{2s|\mathcal{I}}-t)}{\sqrt {\delta_{2s|\mathcal{I}}^{2}-t^{2}}}$, $\beta=\frac{(\delta_{2s|\mathcal{I}}+t)}{\sqrt{\delta_{2s|\mathcal {I}}^{2}-t^{2}}}$, we get

32 $$ \langle Au, Aw\rangle\geq-\sqrt{\delta_{2s|\mathcal{I}}^{2}-t^{2}}. $$

Combining (31) with (32) yields the desired inequality (29). Next, noticing that $Av_{S}=A(v-\sum_{j\geq1}Av_{S_{j}})$, we have

33 $$ \begin{aligned}[b]\|Av_{S}\|_{2}^{2}& = \langle Av_{S}, Av \rangle-\sum_{j\geq1}\langle Av_{S}, Av_{S_{j}}\rangle \\ & \leq \|Av_{S}\|_{2}\|Av\|_{2} +\sum _{j\geq1}\sqrt{\delta_{2s|\mathcal{I}}^{2}-t^{2}} \|v_{S}\|_{2}\| v_{S_{j}}\|_{2} \\ & = \|v_{S}\|_{2} \biggl(\sqrt{1+t}\|Av\|_{2}+ \sqrt{\delta_{2s|\mathcal {I}}^{2}-t^{2}}\sum _{j\geq1}\|v_{S_{j}}\|_{2} \biggr). \end{aligned}$$

According to Lemma A.1 and the setting of $S_{j}$, we have

34 $$ \begin{aligned}[b]\sum_{j\geq1}\|v_{S_{j}} \|_{2,2} & \leq \sum_{j\geq1} \biggl[ \frac{1}{\sqrt{s}}\|v_{S_{j}}\|_{2,1} +\frac{\sqrt{s}}{4}\bigl( \big\| v_{S_{j}}[1]\big\| _{2}-\big\| v_{S_{j}}[s]\big\| _{2}\bigr) \biggr] \\ & \leq \frac{1}{\sqrt{s}}\|v_{S^{C}}\|_{2,1}+\frac{\sqrt{s}}{4} \big\| v_{S_{1}}[1]\big\| _{2} \\ & \leq \frac{1}{\sqrt{s}}\|v_{S^{C}}\|_{2,1}+\frac{1}{4} \|v_{S}\|_{2}. \end{aligned}$$

Substituting (34) into (33) and noticing (28), we also have

$$ (1+t) \|v_{S}\|_{2}\leq \sqrt{1+t}\|Av\|_{2}+ \frac{\sqrt{\delta _{2s|\mathcal{I}}^{2}-t^{2}}}{\sqrt{s}}\|v_{S^{C}}\|_{2,1} +\frac{\sqrt{\delta_{2s|\mathcal{I}}^{2}-t^{2}}}{4} \|v_{S}\|_{2}, $$

that is,

$$ \|v_{S}\|_{2}\leq \frac{\sqrt{\delta_{2s|\mathcal{I}}^{2}-t^{2}}}{\sqrt {s}(1+t)}\|v_{S^{C}} \|_{2,1} +\frac{\sqrt{\delta_{2s|\mathcal{I}}^{2}-t^{2}}}{4(1+t)}\|v_{S}\|_{2}+ \frac {1}{\sqrt{1+t}}\|Av\|_{2}. $$

Let

35 $$ f(t)=\frac{\sqrt{\delta_{2s|\mathcal{I}}^{2}-t^{2}}}{1+t},\quad|t|\leq \delta_{2s|\mathcal{I}}, $$

then it is not difficult to conclude that $f(t)$ has a maximum point $t=-\delta_{2s|\mathcal{I}}^{2}$ in the closed interval $[-\delta_{2s|\mathcal{I}}, \delta_{2s|\mathcal{I}}]$, so for $|t|\leq\delta_{2s|\mathcal{I}}$, we have

36 $$ f(t)=\frac{\sqrt{\delta_{2s|\mathcal{I}}^{2}-t^{2}}}{1+t}\leq\frac{\delta _{2s|\mathcal{I}}}{\sqrt{1-\delta_{2s|\mathcal{I}}^{2}}}. $$

Therefore,

$$ \|v_{S}\|_{2}\leq \frac{\delta_{2s|\mathcal{I}}}{\sqrt{1-\delta _{2s|\mathcal{I}}^{2}}}\frac{1}{\sqrt{s}} \|v_{S^{C}}\|_{2,1} +\frac{\delta_{2s|\mathcal{I}}}{4\sqrt{1-\delta_{2s|\mathcal{I}}^{2}}}\| v_{S} \|_{2} +\frac{1}{\sqrt{1-\delta_{2s|\mathcal{I}}}}\|Av\|_{2}, $$

that is,

$$ \|v_{S}\|_{2}\leq \frac{4\delta_{2s|\mathcal{I}}}{4\sqrt{1-\delta _{2s|\mathcal{I}}^{2}}-\delta_{2s|\mathcal{I}}}\frac{1}{\sqrt{s}}\| v_{S^{C}}\|_{2,1} +\frac{4\sqrt{1+\delta_{2s|\mathcal{I}}}}{4\sqrt{1-\delta_{2s|\mathcal {I}}^{2}}-\delta_{2s|\mathcal{I}}}\|Av\|_{2}. $$

Here, we require

37 $$ 4\sqrt{1-\delta_{2s|\mathcal{I}}^{2}}-\delta_{2s|\mathcal{I}}>0, \qquad \frac{4\delta_{2s|\mathcal{I}}}{4\sqrt{1-\delta_{2s|\mathcal {I}}^{2}}-\delta_{2s|\mathcal{I}}}< 1, $$

which implies $\delta_{2s|\mathcal{I}}^{2}<\frac{16}{41}$, that is, $\delta_{2s|\mathcal{I}}<\frac{4}{\sqrt{41}}\approx0.6246$. □

### Remark 1

Substituting (27) into (25) and (26), we can obtain the constants in Theorem 1.

### Remark 2

Our result improves that of [1], that is, the bound of block RIC $\delta_{2s|\mathcal{I}}$ is improved from 0.4931 to 0.6246.

## Conclusions

In this paper, we gave a new bound on the block RIC $\delta_{2s|\mathcal {I}}<0.6246$, under this bound, every block *s*-sparse signal can be exactly recovered via the $l_{2}/l_{1}$-minimization approach in the noiseless case and is stably recovered in the noisy measurement case. The result improves the bound on the block RIC $\delta_{2s|\mathcal{I}}$ in [1].

## Appendix

### Lemma A.1

*Suppose that* $v\in\mathbb{R}^{N}$ *is split into* *m* *blocks*, $v[1],v[2],\ldots,v[m]$, *which are of length* $d_{1}, d_{2},\ldots,d_{m}$, *respectively*, *that is*,

38 $$ v=[\underbrace{v_{1},\ldots,v_{d_{1}}}_{v[1]}, \underbrace {v_{d_{1}+1},\ldots,v_{d_{1}+d_{2}}}_{v[2]},\ldots, \underbrace{v_{N-d_{m}+1},\ldots,v_{N}}_{v[m]}]^{T}. $$

*Suppose that the* *m* *blocks in* *x* *are rearranged by nonincreasing order for which*

$$ \big\| v[1]\big\| _{2} \geq\big\| v[2]\big\| _{2} \geq\cdots\geq\big\| v[m] \big\| _{2} \geq0. $$

*Then*

39 $$\begin{aligned}[b] \sqrt{\big\| v[1]\big\| _{2}^{2}+\cdots+\big\| v[m] \big\| _{2}^{2}} \leq{}&\frac{\|v[1]\|_{2}+\cdots+\|v[m]\|_{2}}{\sqrt{m}} \\ & + \frac{\sqrt{m}}{4}\bigl(\big\| v[1]\big\| _{2}-\big\| v[m]\big\| _{2} \bigr). \end{aligned}$$

### Proof

See Lemma 6.14 in [30] for the details. □
